# A comparison between different anti-retroviral therapy regimes on soluble inflammation markers: a pilot study

**DOI:** 10.1186/s12981-020-00316-w

**Published:** 2020-10-14

**Authors:** Martina Maritati, Trentini Alessandro, Nunzia Zanotta, Manola Comar, Tiziana Bellini, Laura Sighinolfi, Carlo Contini

**Affiliations:** 1 Section of Infectious Diseases, Department of Medical Sciences, University of Ferrara, 44124 Ferrara, Italy; 2 Department of Biomedical & Specialty Surgical Sciences, University of Ferrara, Ferrara, Italy; 3 Department of Medical Sciences, University of Trieste, Trieste, Italy; 4 Institute for Maternal and Child Health – IRCCS “Burlo Garofolo”, Trieste, Italy; 5 Infectious Diseases Unit, Azienda Ospedaliero-Universitaria di Cona (Ferrara), Ferrara, Italy

**Keywords:** HIV, Virological suppression, Antiretroviral therapy, Immune activation, Chronic inflammation

## Abstract

**Background:**

Although HIV-related deaths have decreased dramatically following the introduction of antiretroviral therapy (ART), HIV infection itself causes increased morbidity and mortality for both non-AIDS-related events or chronic inflammation and immune activation. The use of certain antiretroviral drugs can contribute to this process.

**Methods:**

We investigated 26 potential biomarkers in serum samples from HIV-1 infected patients virologically suppressed under ART. The main objective of our study was to evaluate if virological suppression achieved with a triple drug regimen containing tenofovir disoproxil fumarate co-formulated with emtricitabine (TDF/FTC) as backbone, could correlate with a better immunological and inflammatory profile in relation to the third class of antiretroviral drug administered. The eligible patients were then divided into 3 groups in relation to the third drug associated with TDF/FTC: nucleoside reverse transcriptase inhibitors (NNRTI) (Group 1, n = 16), protease inhibitors (PI) (Group 2, n = 17) and integrase inhibitors (INI) (Group 3, n = 16).

**Results:**

Inflammatory cytokines and chemokines were more represented in Group 2 than in Group 3 (IL-1Ra, *p* = 0.013; IL-12p70 *p* = 0.039; TNF-α *p* = 0.041; IL-8, *p* = 0.027; MIP1 β, *p *= 0.033). Eotaxin showed lower levels in Group 1 compared to Group 2 (*p *= 0.010), while IP-10 was significantly lower in Group 1 compared to both Group 2 and Group 3 (*p* = 0.003 and p = 0.007, respectively).

**Conclusions:**

Our results seem to discourage the administration of PI as a third drug in a virologically effective antiretroviral regimen, as its use is linked to the detection of higher levels of pro-inflammatory mediators in comparison with INI and NNRTI.

## Background

The introduction of antiretroviral therapy (ART) has contributed to the reduction of AIDS-related mortality among HIV-positive patients [1] and to the induced chronicity of HIV infection. Triple drug regimens that include two nucleos(t)ide reverse transcriptase inhibitors (NRTI) as the backbone plus a base agent of another class, protease inhibitors (PI), integrase inhibitors (INI) or nucleoside reverse transcriptase inhibitors (NNRTI) [2], have been shown to control viral replication [3] and represent the gold standard for the treatment of HIV infection in both antiretroviral-naïve and antiretroviral experienced patients. Now, these patients live longer, although with a higher prevalence of non-infectious comorbidities including cardiovascular disease, diabetes, renal dysfunction and liver damage. Although these comorbidities are quite common among the general population [4], they are increasingly frequent in patients with ART [5] more likely due to the inflammatory process related to HIV and the adverse pharmacological effects of ART [6–11].

In this regard, the international guidelines on the management of HIV-positive patients recommend a change in lifestyle (smoking cessation, changes in diet and physical activity) and, in case of high LDL and/or hypertriglyceridemia values, the administration of statins. In addition to these measures, it is highly recommended to move to an antiretroviral regimen that limits the impact on lipid metabolism, suggesting the adoption of a therapy “tailored” to the needs of the patient.

Clinical studies have also shown that the transition from therapies such as PI or NNRTI (efavirenz) to integrase inhibitors (INI) (e.g. raltegravir, dolutegravir) leads to an improvement in the lipid profile, inflammatory pattern, and chronic immune activation. In fact, HIV infection combined with ART is directly associated with immune activation [12, 13], even in virologically suppressed patients. It is known, indeed, that the dysregulation of inflammatory processes induced by HIV occurs through multiple pathways [14], including microbial translocation [15].

To better understand this phenomenon and to optimize therapeutic management, some authors have recently been stimulated to seek and validate new immunological and inflammatory predictive biomarkers.

So far, most studies examining changes in levels of inflammatory biomarkers in HIV-infected individuals have been limited by small study populations, cross-drawings and/or a small number of biomarkers [16]. In this regard, recent technical advances, including the development of multiplexed cytokine tests, have contributed to a more efficient measurement of multiple inflammatory biomarkers [17].

In this study, we investigated 26 potential biomarkers, linked to systemic inflammation, immune activation and senescence in HIV patients under ART for at least one year and virologically suppressed (HIV-RNA < 20 copies/ml) for at least 6 months. All the enrolled patients were treated with an ART triple drug regimen containing tenofovir disoproxil fumarate co-formulated with emtricitabine (TDF/FTC) as backbone. The main aim of our study was to assess whether virological suppression can be related to a better immunological and inflammatory profile, in relation to the third antiretroviral drug administered.

## Methods

### Study patients and design

Forty-nine caucasian HIV-1 positive patients aged ≥ 40 years, who have taken ART for at least one year, were enrolled at the Infectious Diseases Department (HIV Section) University-Hospital of Ferrara. Strict inclusion criteria were considered: (1) virological suppression for at least 6 months obtained with a triple drug regimen containing TDF/FTC as a backbone; (2) Cardiovascular risk (CVR) ≥ 7.5%, calculated according to the Atherosclerotic Cardiovascular Disease (ASCVD) algorithm of American College of Cardiology/American Heart Association (ACC-AHA) [18], in the absence of a regimen with statin and / or aspirin. The presence of comorbidities (chronic inflammatory diseases, neoplasms, diabetes, obesity, etc.) and co-infections represented a strict exclusion criterion.

Eligible patients were then divided into groups according to the third drug associated with TDF/FTC at the time of enrollment: NNRTI (efavirenz) (Group 1, n = 16), PI (atazanavir/r or darunavir/r) (Group 2, n = 17) and INI (raltegravir) (Group 3, n = 16).

This study is in line with the Code of Ethics of the World Medical Association (Declaration of Helsinki) and was conducted according to the guidelines for Good Clinical Practice (European Medicines Agency). The study was approved by The Local Ethic Committee and written informed consent was obtained from each patient prior to inclusion in the study.

### Serum sample collection

Serum samples were obtained from the 49 enrolled subjects. The whole blood of each subject was collected in a covered tube without anticoagulants and left to clot undisturbed at room temperature for 20 min. The clot was then removed by centrifuging at 1500 × g for 10 min in a refrigerated centrifuge. After centrifugation, the serum was immediately transferred to a clean polypropylene tube. All sera were maintained at − 80 °C until cytokines analysis.

### Chemokines and cytokines analysis

The main outcome measures were the quantification of cytokine concentrations and growth factors in biological samples based on magnetic bead multiplex immunoassays (Bio-Plex, BIO-RAD Laboratories, Milano, Italy). Luminex multiplex panel technology was used for simultaneous measurement of a panel of 26 analytes including cytokines, chemokines and growth factors (IL-1, IL-2, IL-1ra, IL-4, IL-5, IL-6, IL-9, IL-10, IL-12p70, IL-13, IL-15, TNF-α, IL-17, IL-18, IFN-γ, MIP1α, MIP1β, IL-8, IP-10, RANTES, MCP-1, GM-CSF, G-CSF, IL-7, VEGF, PDGF-bb) according to the method of Comar et al. 2014. Briefly, 50 μl of diluted serum samples (1:4) and reaction standards were added, in duplicate, to a 96 multiwells plate containing analyte beads followed by incubation for 30 min at room temperature. After washing, the antibody-biotin reporter was added and incubated for 10 min with streptavidin–phycoerythrin. Cytokines levels were determined using the Bio-Plex array reader (Luminex, Austin, TX). The Bio-Plex Manager software automatically optimized the standard curves and returned the reading data as Median Fluorescence Intensity (MFI) and concentration (pg/mL). An ELISA set (Quantikine ELISA-Human CCL5/Rantes immunoassay, RnD system, Minneapolis, MN) with a mean minimum detectable dose of 2.0 pg/ml was used as confirmatory test according to manufacturer’s instruction [17].

### Statistical analysis

The normality of distribution of continuous variables was assessed by Kolmogorov–Smirnov test; since variables were not normally distributed, group comparisons were made by Kruskall-Wallis followed by the Mann–Whitney U test corrected for multiple comparisons (Bonferroni). To correct for possible confounding factors, such as age, sex, smoke and disease duration, group comparisons were performed on natural logarithm-transformed variables by ANCOVA, including the variables listed as covariates. The Spearman’s Rank Test was used to analyze bivariate correlations. The categorical variables were compared by Chi Square Test.

Data analysis was performed using SPSS Statistics for Windows, version 21.0 (SPSS, Inc., Chicago, IL, USA). Two-tailed probability values < 0.05 were considered statistically significant.

## Results

The demographic and clinical characteristics of the population studied are summarized in Table 1.

**Table 1:** Demographic and clinical characteristic of the study population

	Group 1	Group 2	Group 3
Age (years)	49.7 (45.9–57.0)	52.9 (47.4–57.1)	53.7 (45.3–61.6)
CD4 +	857 (669–1045)	727 (527–926)	701 (545–857)
Sex (female, %)	44.4	50.0	33.3
Smoking status (smokers, %)	55.6	50.0	50.0
Disease Duration (years)	16.0 (11.3–20.7)	15.5 (11.6–19.5)	10.4 (8.7–12.2)
Triglycerides (mg/dL)	150 (83–217)	134 (91–176)	126 (103–149)
Total cholesterol (mg/dL)	220 (193–247)	210 (185–235)	229 (210–248)

Continue variables are expressed as mean (95% Confidence Interval). Categorical variables are expressed as percentage

Of the 49 patients enrolled, 14 were women (28.57%) and 35 were males (71.43%).

The values of triglycerides were high (> 150 mg/dL) in 33%, 35% and 31% of patients from NNRTI, PI and INI group, respectively. The frequencies were not different between the three groups (chi-square (2) = 0.061, *p* = 0.970).

Total cholesterol was high (> 200 mg/dL) in 60%, 53% and 69% of patients from NNRTI, PI and INI group, respectively. Also in this case, the frequencies of high total cholesterol levels were not different between the three groups (chi-square (2) = 0.863, *p* = 0.650).

A first exploratory statistical analysis showed that one patient, belonging to Group 1, showed extreme values (absolute value of z-score > 1.6) for 8 (30.7%) of the 26 cytokines analyzed and, consequently, was excluded from the analysis.

Although statistical significance was achieved for only few mediators, probably because of the small sample size, our results showed that 7 of the 26 immune mediators (18.2%), analyzed between the different groups, are influenced by the type of third drug administered (Additional file 1. Figure S1).

As for the group of innate immunity mediators, some traditionally inflammatory cytokines and chemokines were more represented in PI-treated serum samples with higher levels than those treated with INI (IL-1Ra, p = 0.013; IL-12p70 p = 0.039; TNF-α *p* = 0.041; IL-8, *p* = 0.027; MIP1 β, *p* = 0.033). Eotaxin showed lower levels in Group 1 compared to Group 2 (p = 0.010), while IP-10 was significantly lower in Group 1 compared to both Group 2 and Group 3 (*p* = 0.003 and *p* = 0.007, respectively). Of note, almost 47% of patients in both NNRTI and PI groups had abnormal cytokine values (|z-score|> 1.6), whereas 25% in the INI group showed this behavior (Additional file 1. Table S2). However, the difference in frequencies between groups did not reach statistical significance (Chi-square (2) = 2.135, *P* = 0.344).

As shown in Table 2, statistically significant correlations between disease duration and levels of some immune mediators were found in the overall population.

**Table 2:** Spearman’s correlation coefficients between Interleukins levels and HIV diseases duration

Interleukin	r	*P* value
IL-1ra	0.434	0.002
IL-7	0.371	0.010
IL-8	0.321	0.028
IL-12	0.295	0.044
G-CSF	0.325	0.026
IFNg	0.314	0.032
MCP-1	0.330	0.023
TNFa	0.402	0.005

**Fig. 1:**
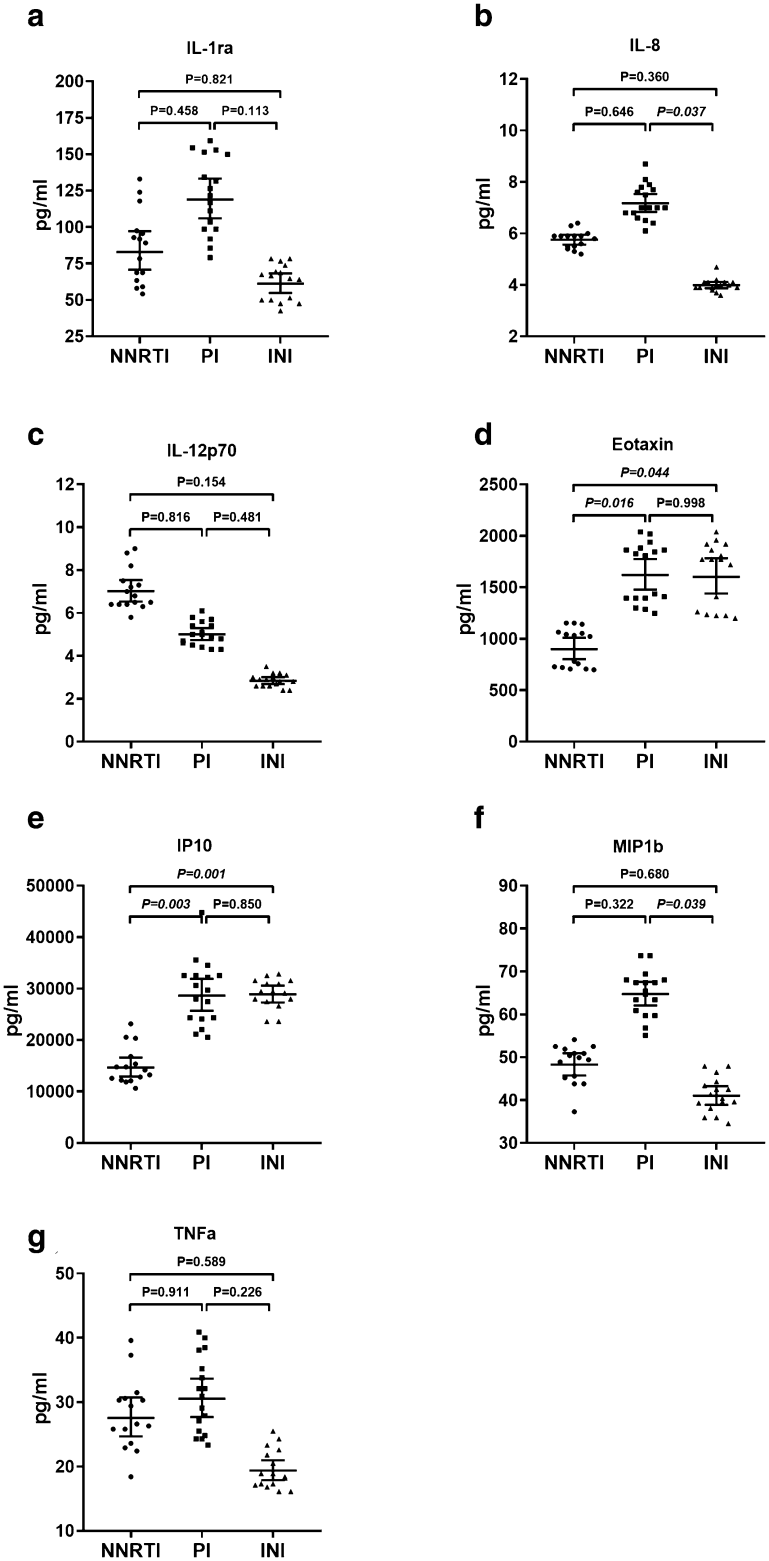
Predicted values of cytokines (pg/ml) corrected for covariates (age, sex, smoking status and disease duration) by the ANCOVA approach. Comparisons between groups were performed with the Sidak post-hoc test correction for multiple comparisons. Values of covariates appearing in the model: age = 52.4 years; sex = 0.67; smoking status; 0.56; HIV duration = 13.9 years. **a** IL1ra; **b** IL-8; **c** IL-12; **d** Eotaxin; **e** IP10; **f** MIP1b; **g** TNF-α. Bars and error bars denote geometric mean and 95% confidence interval

The univariate analysis (ANOVA) showed that there were no statistical differences in disease duration between groups, although INI patients showed a tendency to have lower disease duration values. In order to correct the cytokine values for possible confounding factors (age, sex, smoking status, duration of disease), we used an ANCOVA approach on natural logarithm-transformed variables. As summarized in Additional file 1. Table S1 and Fig. 1, some of the immune mediators tested were affected by confounding factors, in particular IL-1ra, IL12p70 and TNF-α which resulted no longer different between the groups. On the contrary, the other mediators were not affected by the correction.

## Discussion

Although HIV-related deaths have declined dramatically since the introduction of ART, HIV infection is becoming increasingly chronic and people infected with HIV continue to experience raised morbidity and mortality often due to events unrelated to AIDS [19]. In fact, due to the inability of antiretroviral drugs to eradicate the virus from infected reservoir cells, treatment of HIV infection requires permanent systemic therapy. However, even when successfully treated, HIV patients still show higher incidence of age-associated co-morbidities than non-infected individuals. Chronic Immune Activation and Senescence (CIADIS), is a process characterized by a progressive decline of immune system function, usually detected by the expression of cellular or soluble markers derived from innate or adaptive immune responses. Immune activation is associated with progression of HIV disease and increased morbidity and mortality in HIV-infected patients despite ART [20]. In this regard, the validation of a “CIADIS score” based on activation, senescence, and differentiation markers, could help physicians to identify patients at high risk for non-AIDS-related comorbidities [20]. In fact, it is striking evident that cardiovascular diseases are one of the leading non-AIDS causes of death among HIV-positive subjects [21].

Although not fully understood, the probable mechanism involves both chronic inflammation, CD4 cell depletion, endothelial dysfunction and atherosclerosis [22]. It is undoubted that the worst lipid profile (e.g. higher total cholesterol, triglycerides, and LDL-cholesterol than recommended values) that accompanies the HIV affected patients may play a significant role in this [23]. However, in our study the analysis conducted on total cholesterol and triglycerides did not show statistically significant differences between the three groups. This data contrasts with what has been reported in some papers, according to which INI are more “lipid friendly” [24, 25].

Although ART has significantly improved both the quality and lifespan of patients, the life expectancy of treated patients is even shorter than that of uninfected individuals. In particular, while ART may counteract some features of HIV-associated immunosenescence, several anti-HIV drugs may themselves help to amplify other aspects of immune ageing and chronic inflammation [26].

The analysis conducted in the present study reveals that some categories of antiretroviral drugs emphasize the residual inflammation, partly linked to HIV infection itself [12, 13].

According to our findings, some inflammatory cytokines of innate immunity (Additional file 1. Figure S1a, c, g) and two pro-inflammatory chemokines (Additional file 1. Figure S1b, f) are more represented in patients taking PI than those receiving INI.

There are several studies comparing the effects of specific antiretrovirals and antiretroviral combinations in ART-naïve individuals initiating their first ART regimen. In one recent ART initiation trial, INI appear to reduce inflammation to a greater degree than NNRTI; however, in the same study, it is not clear if there are beneficial effects on inflammation resulting from treatment with INI compared to PI or between PI and NNRTI [27].

It is plausible that INI may decrease inflammation and immune activation more than other antiretroviral classes, as INI may concentrate at higher levels in enterocytes [28], which is important because HIV infection results in massive depletion of immune cells within the gastro-intestinal tract and the resultant microbial translocation may be an important driver of immune activation in HIV. In line with this, we found that INI patients had a tendency to show a lower frequency of extreme cytokine levels when compared to the other groups.

However, in contrast with the above statement, our results show that the IFN-γ induced protein 10 (IP-10 or CXCL-10) and eotaxin (also known as C–C chemokine ligand 11, CCL1) are significantly lower in patients treated with NNRTI compared to those belonging to the other groups (Fig. 1e, d). This finding appears relevant especially for IP-10, considering that this mediator is known to be one of the first chemokines to increase following HIV infection [29] and it is involved in immune cell trafficking to inflammatory sites. In particular, the continued production of IFN-γ in the lymphoid organs is responsible for a prolonged increase in IP-10 during chronic HIV infection, even in patients taking ART. In fact, IP-10, by increasing the susceptibility of naive T CD4 + T lymphocytes to HIV infection, improves the production (constitution) of HIV reserves.

The present study is not without limitations, first of all with regard to the small sample size, followed by a cross sectional design and the lack of comparison of the immune mediators tested with other conventional inflammatory or procoagulant markers such as CRP and D-dimer respectively [30]. The lack of a complete set of biomarkers hamper an exhaustive evaluation of the cardiovascular risk and the real inflammatory profile among the three groups and represents the main limitation of the present study.

However, although with limitations, our data seem to highlight the importance of how the choice of the third drug in an antiretroviral regimen can promote the imbalance of inflammation-related immune mediators. In particular, our results seem to discourage the administration of PI as a third drug in a virologically effective antiretroviral regimen, as its use is linked to the detection of higher levels of pro-inflammatory mediators than INI and NNRTI.

Further studies in larger cohorts are needed to confirm the results found, to compare it to conventional markers of inflammation, and to provide their usefulness as a significant clinical tool to help clinicians in choosing a “tailored” antiretroviral regimen.

## Supplementary information

**Additional file 1: Figure S1.** Differences in cytokine concentrations (pg/ml) measured in patients following the therapy simplification regimen. **Table S1.** Crude and adjusted means of ILs values determined in study population. **Table S2.** Proportion of outliers found in each group.

## Data Availability

The datasets used and/or analysed during the current study are available from the corresponding author on reasonable request.
