# Supplementary material for: A comparison between different anti-retroviral therapy regimes on soluble inflammation markers: a pilot study
Source: AIDS Res Ther. 2020 Oct 14;17:61. doi: 10.1186/s12981-020-00316-w (PMC7558668; doi:10.1186/s12981-020-00316-w)
Supplement: Supplementary file 1 — Additional file 1: Figure S1. Differences in cytokine concentrations (pg/ml) measured in patients following the therapy simplification regimen. Table S1. Crude and adjusted means of ILs values determined in study population. Table S2. Proportion of outliers found in each group. [file 12981_2020_316_MOESM1_ESM.docx]

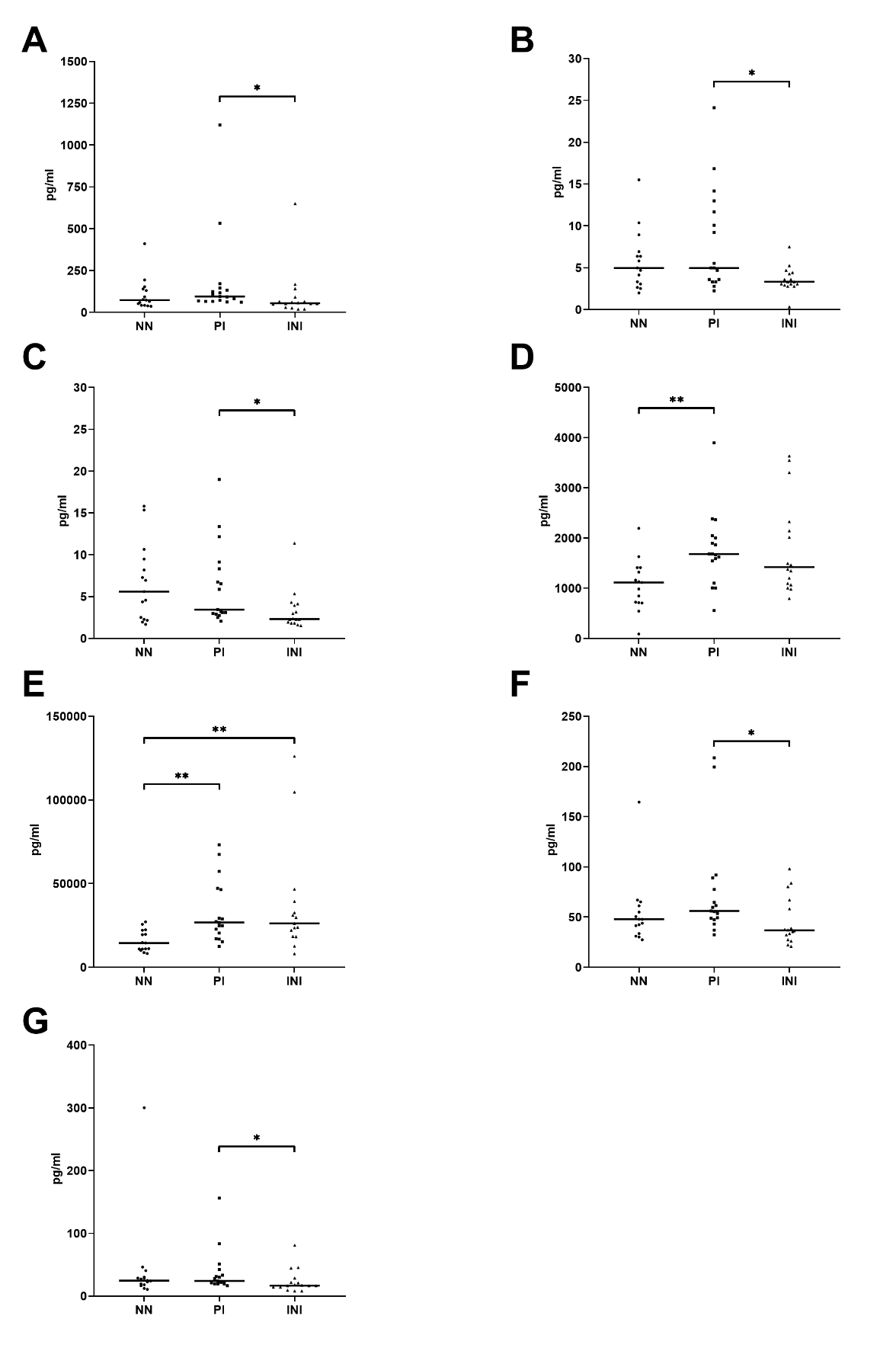


**Supplementary Figure 1.** Differences in cytokine concentrations (pg/ml) measured in patients following the therapy simplification regimen. Statistical comparisons between groups were performed with the Kruskall-Wallis test followed by Mann-Whitney U test corrected for multiple comparisons. Panel A: IL1ra; Panel B: IL-8; Panel C: IL-12; Panel D: Eotaxin; Panel E: IP10; Panel F: MIP1b; Panel G: TNF-α. * P<0.05; ** P<0.01.

| **Table S1. Crude and adjusted means of ILs values determined in study population** | | | | | | | | |
| --- | --- | --- | --- | --- | --- | --- | --- | --- |
|  | **Crude geometric means (95% CI)** | | | | **Adjusted means (95% CI)** | | | |
| **Interleukin** | **Group 1** | **Group 2** | **Group 3** | **P value** | **Group 1** | **Group 2** | **Group 3** | **P value** |
| IL-1ra | 82.8 (56.4-121.5) | 118.7 (79.4-177.7) | 61.1 (38.1-97.5) | 0.017 | 80.5 (52.6-123.0) | 117.9 (79.6-174.7) | 63.2 (41.3-96.6) | 0.105 |
| IL-8 | 5.7 (4.4-7.6) | 7.2 (5.5-9.3) | 4.0 (3.0-5.2-) | 0.030 | 5.7 (4.2-7.7) | 7.1 (5.3-9.3) | 4.1 (3.0-5.5) | 0.042 |
| IL-12p70 | 7.3 (4.3-12.3) | 5.0 (3.0-8.3) | 2.5 (1.7-4.8-) | 0.020 | 6.8 (3.8-12.2) | 4.9 (2.9-8.6) | 2.9 (1.6-5.2) | 0.149 |
| Eotaxin | 898 (671-1202) | 1620 (1232-2130) | 1603 (1208-2125) | 0.009 | 912 (679-1226) | 1626 (1237-2137) | 1573 (1170-2113) | 0.011 |
| IP10 | 14646 (10950-19591) | 28679 (21822-37689) | 28906 (21611-38664) | 0.001 | 13723 (10215-18430) | 27639 (21018-36362) | 32179 (23717-43624) | 0.0004 |
| MIP1b | 48.3 (37.6-62.1) | 64.7 (51.2-81.8) | 41.0 (32.2-52.2) | 0.0034 | 49.1 (37.7-63.9) | 64.8 (50.8-82.8) | 40.4 (30.9-52.6) | 0.039 |
| TNF-alpha | 31.0 (21.7-44.3) | 30.6 (21.6-43.2) | 19.4 (13.5-27.7) | 0.041 | 26.7 (18.6-38.2) | 30.8 (22.1-42.9) | 19.7 (13.8-28.3) | 0.212 |
| Covariates appearing in the model: age = 52.4 years; sex = 0.67; smoking status; 0.56; HIV duration = 13.9 years. | | | | | | | | |

| **Table S2. Proportion of outliers found in each group.** | | | |
| --- | --- | --- | --- |
|  | **NNRTI (%)** | **PI (%)** | **INI (%)** |
| **Normal** | 53.3 | 52.9 | 75.0 |
| **Outlier** | 46.7 | 47.1 | 25.0 |
| Outliers have been detected by: 1) converting cytokine values into z-scores; 2) observing if the absolute value of z-score was >1.6. If so, it was considered an outlier. Chi-square test between the groups was not significant. | | | |
